# Supplementary figures and images for: Anti-inflammatory effect of the combined treatment of LMT-28 and kaempferol in a collagen-induced arthritis mouse model
Source: PLoS One. 2024 Jul 31;19(7):e0302119. doi: 10.1371/journal.pone.0302119 (PMC11290667; doi:10.1371/journal.pone.0302119)

Whole membrane of blots in western blotting

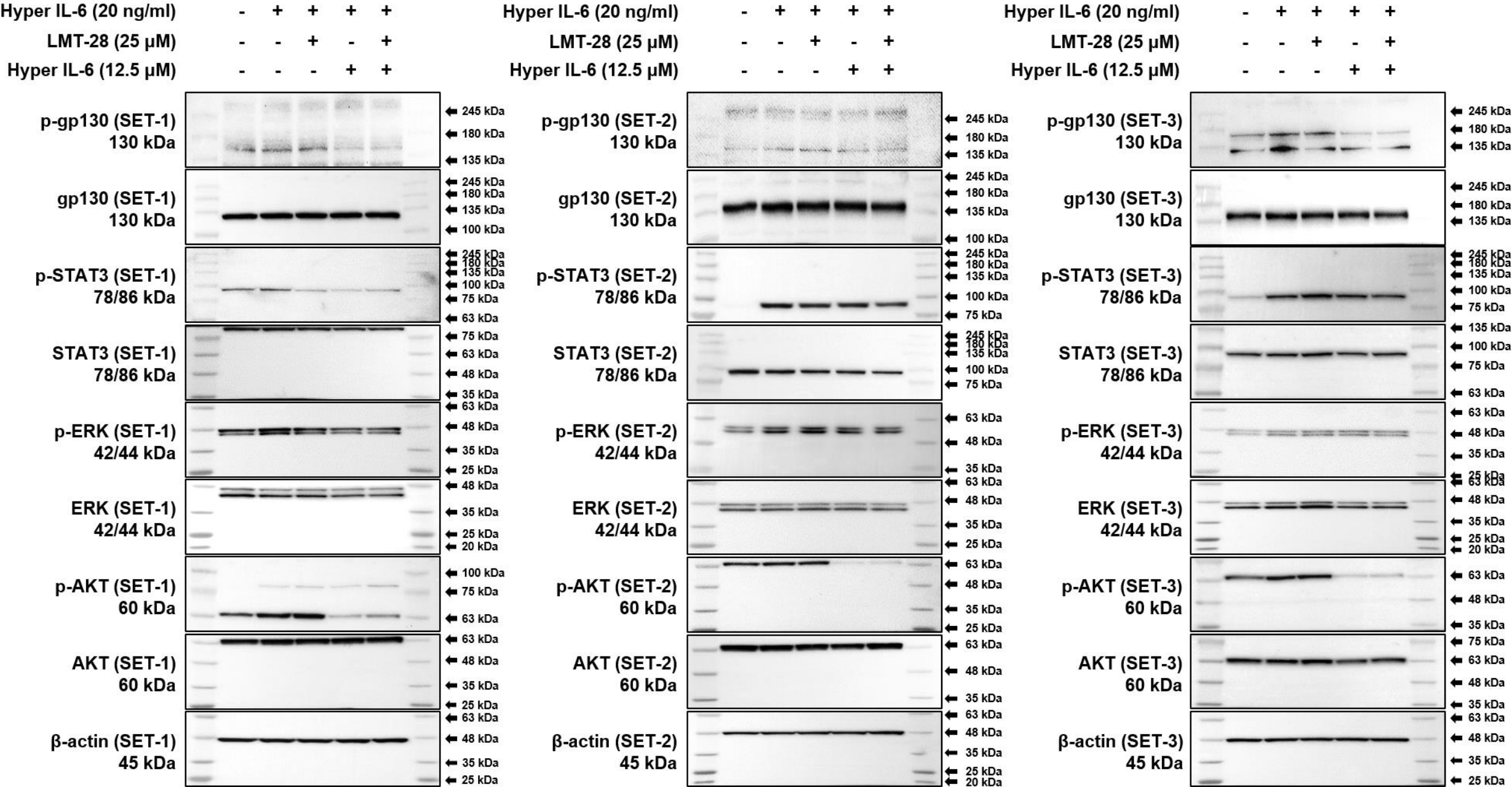

Supplement: S1 Raw images — (PDF) [file pone.0302119.s001.pdf]
